# Case Report: Clinical Characteristics and Outcomes of HIV Positive Patients With Metastatic Prostate Cancer Treated With Immunotherapy: A Case Series and Literature Review

**DOI:** 10.3389/fonc.2022.910115

**Published:** 2022-07-07

**Authors:** Dame Idossa, Terence Friedlander, Channing J. Paller, Charles J. Ryan, Hala T. Borno

**Affiliations:** ^1^ Division of Hematology/Oncology, Department of Medicine, University of California, San Francisco (UCSF), CA, United States; ^2^ UCSF Helen Diller Family Comprehensive Cancer Center, San Francisco, CA, United States; ^3^ Johns Hopkins Sidney Kimmel Comprehensive Cancer Center, Baltimore, MD, United States; ^4^ Department of Medicine, Zuckerberg San Francisco General Hospital, San Francisco, CA, United States; ^5^ Division of Hematology/Oncology and Transplantation, Department of Medicine, University of Minnesota, Minneapolis, MN, United States; ^6^ Prostate Cancer Foundation, Santa Monica, CA, United States

**Keywords:** HIV, immunotherapy, pembrolizumab, prostate cancer, PD-1 inhibitor, checkpoint blockade, mCRPC

## Abstract

**Background:**

The treatment of metastatic prostate cancer has been revolutionized with the advent of many targeted therapies, including immunotherapy. Pembrolizumab has demonstrated benefit in the treatment of certain patients with docetaxel-refractory metastatic castrate-resistant prostate cancer (mCRPC). However, extrapolation of these data to patients with HIV is limited, as these patients are conventionally excluded from therapeutic clinical trials. This study aims to develop a better understanding of the clinical outcomes of HIV positive patients with prostate cancer treated with immunotherapy. A review of the literature is conducted on the use of immunotherapy in HIV positive patients with prostate cancer, and a summary is presented of two clinical cases from a single institution.

**Methods:**

This is a retrospective case report of 2 patients diagnosed with prostate cancer and HIV who received treatment with pembrolizumab. Quantitative analysis was performed to summarize patient demographics, clinical history, and outcomes.

**Results:**

Two patients with mCRPC and HIV on **highly active antiretroviral therapy** were identified. Both individuals had biochemical and radiographic response to treatment with pembrolizumab. The duration of response for individual 1 is >31 months and 14 months for individual 2. Neither patient had immune-related adverse events or decreased suppression of their HIV infection. One patient died from disease progression after 14 months of treatment and the other remains on treatment with pembrolizumab to date.

**Conclusion:**

In this small case series, pembrolizumab appears to be a safe and effective treatment option for HIV positive patients with metastatic prostate cancer.

## Introduction

It is well established that HIV infection and the resulting immune suppression can lead to an increased risk of several cancers (1). People with HIV have about 500 times the risk of developing Kaposi sarcoma, 12 times the risk of developing non-Hodgkin lymphoma, and three times the risk of developing cervical cancer. HIV positive individuals are also at increased risk of developing other malignancies, including cancers of the anus, liver, head and neck, lung, and Hodgkin lymphoma (1, 2). Fortunately, with the substantial advancements in highly active antiretroviral therapies, the life expectancy of individuals with HIV is now effectively equal to those without HIV infection (3). Despite these advancements, however, persons with HIV continue to have increased risk of developing cancer compared to the general population. As these individuals live longer, the risk of developing malignancies that are common in the general population also increases.

Prostate cancer is now the second most common neoplasm among the elderly with HIV after lung cancer (4). Men with prostate cancer and well-controlled HIV seem to have clinical presentations and outcomes similar to those without HIV infection, including with regard to surgical and post-operative outcomes (4–6). Furthermore, HIV positive men with prostate cancer have recurrence-free survival outcomes similar to those of the general population (6). However, due to the frequent exclusion of HIV positive men from clinical trials, there has been a knowledge gap with regard to the efficacy of novel therapies, such as immunotherapy, in treating them.

Checkpoint inhibitors – such as inhibitors of cytotoxic T lymphocyte associated protein 4 (CTLA-4), programmed cell death protein 1 (PD-1), and programmed cell death protein 1 ligand (PD-L1) – have revolutionized the approach to treatment of many cancers. For example, pembrolizumab has demonstrated efficacy in select men with docetaxel-refractory mCRPC (7). In general, checkpoint inhibitors work by inhibiting the immune escape mechanisms of cancer cells. In individuals who have underlying systemic conditions affecting the immune system, the efficacy of immunotherapy has not been well defined and extrapolation of these data to individuals with HIV has been limited due to their frequent exclusion from therapeutic clinical trials due to safety concerns. There has also been concern that immunosuppressed individuals may not have sufficient underlying T-cell immunity to benefit from immunotherapy. This has resulted in a paucity of data regarding the efficacy and tolerability of immunotherapy in HIV-positive individuals with prostate cancer.

Data shows that upregulation of PD-1 in CD8 T cells may mediate T-cell exhaustion and lead to progression of HIV (8). This suggests that PD-1 checkpoint inhibition may help control HIV infection, however to date this has not yet been formally studied. A retrospective analysis of 17 HIV individuals across two institutions evaluating the efficacy and safety of checkpoint inhibitors (9) included HIV positive individuals with lung cancer, hepatocellular carcinoma, anal cancer, renal cell carcinoma, non-Hodgkin’s lymphoma, and advanced basal cell carcinoma. The authors of the study concluded that checkpoint inhibitors used to treat these malignancies had comparable efficacy and tolerability and did not adversely affect control of HIV.

The largest study to date on the use of checkpoint inhibitors for treatment of advanced malignancies in individuals with HIV is a prospective open-label, nonrandomized, phase 1 multicenter trial conducted across seven institutions (10). The trial enrolled 30 HIV positive individuals with the following malignancies: Kaposi sarcoma, non-Hodgkin lymphoma, anal cancer, squamous skin cancer, adenoid cystic carcinoma, bladder cancer, cholangiocarcinoma, hepatocellular carcinoma, non-small cell lung cancer, pancreatic cancer, papillary urothelial carcinoma, prostate cancer (only one patient), sarcomatoid lung cancer, and tonsillar cancer. Individuals on this trial were treated with pembrolizumab. The study demonstrated the safety of pembrolizumab in HIV positive individuals with cancer. All individuals from that trial were on ART and had controlled HIV infection as defined by the US department of health and human services. The CD4 positive T-cells remained stable throughout the treatment course. Clinical benefit was demonstrated in those with lung cancer, non-Hodgkin lymphoma, and Kaposi sarcoma.

To date, very little is known about the benefit and safety of PD-1 checkpoint immunotherapy specifically in the treatment of advanced prostate cancer among HIV positive patients. The case series presented here aims to describe the clinical outcomes of two HIV positive men from a single institution who were treated with pembrolizumab.

## Material and Methods

A review was conducted of electronic medical records of patients who were diagnosed with metastatic prostate cancer and HIV at the University of California San Francisco (UCSF) Helen Diller Family Comprehensive Cancer Center. Of the identified cases, a search was made for those who received treatment with pembrolizumab. Data were obtained from clinical notes, pathology reports, and molecular reports. Response evaluation criteria in solid tumors (RECIST) and Prostate Cancer Working Group 3 criteria were used to evaluate serologic and radiologic response to therapy. Patients were categorized as responders to therapy if they achieved complete response, partial response, or stable disease as per the RECIST criteria.

Study data were collected and managed using REDCap electronic data capture tools hosted at UCSF (11, 12). Small sample size precluded more granular stratification and regression analysis. All study procedures were approved by the UCSF Institutional Review Board.

## Results

**Figure 1:**
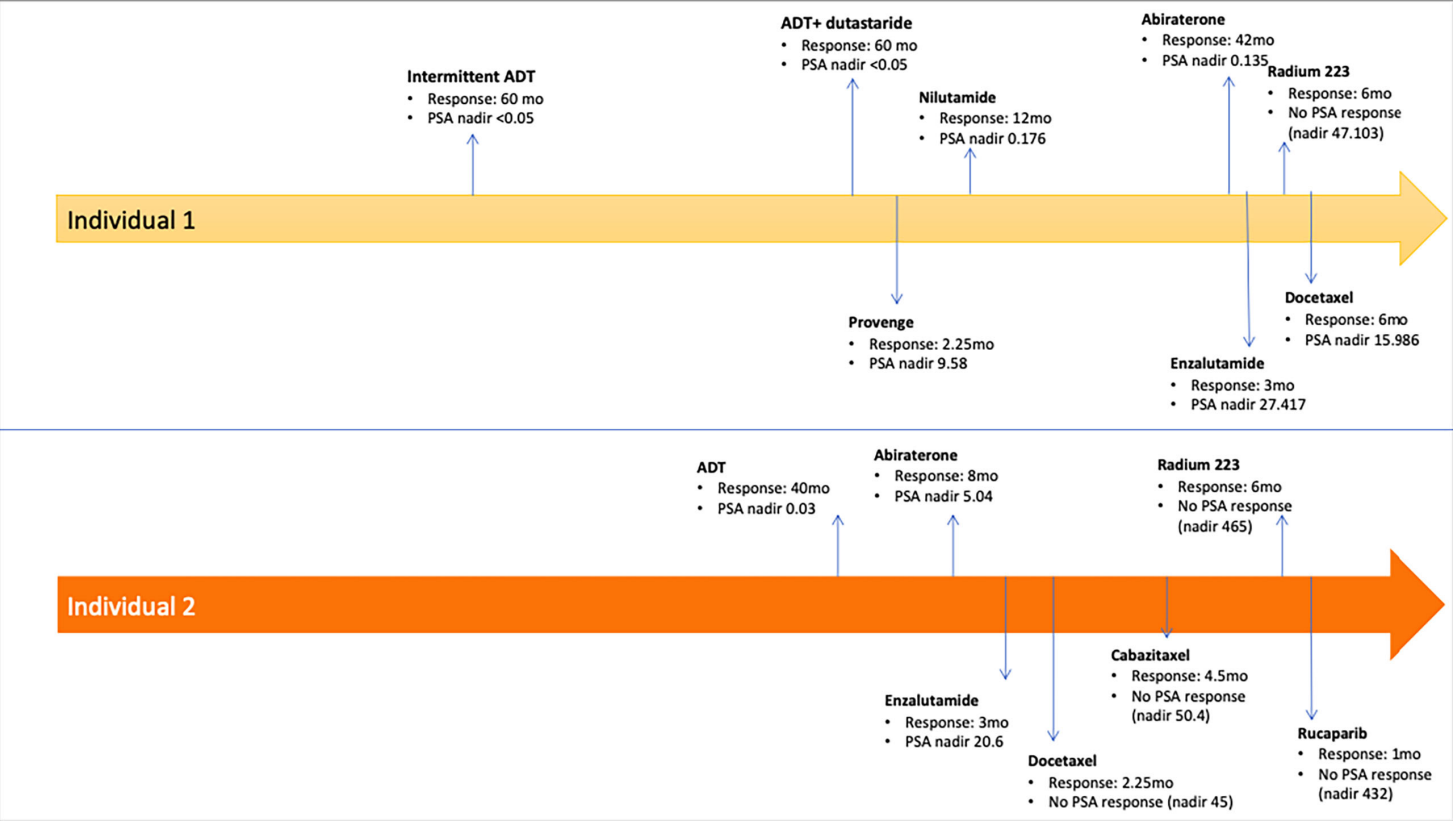
Timeline of Treatments Received Prior to Pembrolizumab.

Two men with HIV and mCRPC to bone and lymph nodes, who were treated with pembrolizumab were identified. Both individuals were heavily pretreated with each having received seven or more prior lines of systemic therapy (**Figure 1**). The clinical characteristics of these men and their response to pembrolizumab are reported in **Table 1**. Individual 1 was diagnosed with HIV at age 36 and developed *denovo* metastatic prostate cancer at age 52. His family history was notable for colon cancer in a brother who died from complication related to this diagnosis at age 52. He also had a family history of unspecified type of cancer in a father and a brother. He had no known family history of prostate cancer. Individual 2 was diagnosed with HIV at age 58 and localized prostate cancer at age 63, which progressed to metastatic disease by age 70. His family history was notable for lung cancer in his father, diagnosed at age 62 and prostate cancer in his two of his brothers (age of diagnosis unknown). Information regarding germline testing of the family members was not available.

**Table 1:** Patient Characteristics of Case Series of HIV Positive Patients with Metastatic Prostate Cancer Treated with Pembrolizumab.

Individual	Age at Prostate Cancer Diagnosis	Time to Prostate Cancer Diagnosis Post-HIV Diagnosis (years)	Prostate-specific Antigen at Start of Immunotherapy (ng/mL)	Prostate-specific Antigen Nadir on Immunotherapy(ng/mL)	RECIST v.1.1Response	Duration of Therapy (months)	Total number of Pembrolizumab cycles received
1	52	16	81	30	Partial	31	36
2	63	5	846	337	Stable	14	19

Both patients were receiving highly active antiretroviral therapy (HAART) for the entire duration of treatment with pembrolizumab. Individual 1 was on Atripla (efavirenz-emtrictabine-tenofovir) with Raltegravir and individual 2 was on Descovy (emtricitabine/tenofov alafenam) with dolutegravir. The median CD4 count at initiation of pembrolizumab was 261 cells/*μ*l (range 170-353 cells/*μ*l). The HIV RNA level was below detection threshold for both patients. Neither patient had known prior complications from the HIV infections and were on HAART for the duration of their prostate cancer therapy. Individual 1 did not have any known germline mutations and somatic next generation sequence (NGS) testing only showed a mutation in AR T878A with 1.3% allele frequency. The microsatellite instability (MSI) status and tumor mutational burden (TMB) was undetermined on their NGS testing. Individual 2 had a variance of unknown significance (VUS), c.2607+5G>A (intronic), in RET gene, a somatic BRCA2 T3310fs*17 mutation with 2.2% allele frequency, stable microsatellite status, low TMB of 4 mutations/mb, TP53 R248W, and TMPRSS2 fusion (TMPRSS(NM_005656)-ERG(NM_004449) mutations. Programmed cell death ligand 1 (PDL-1) testing by immunohistochemistry (IHC) was not performed for both patients.

**Figure 2:**
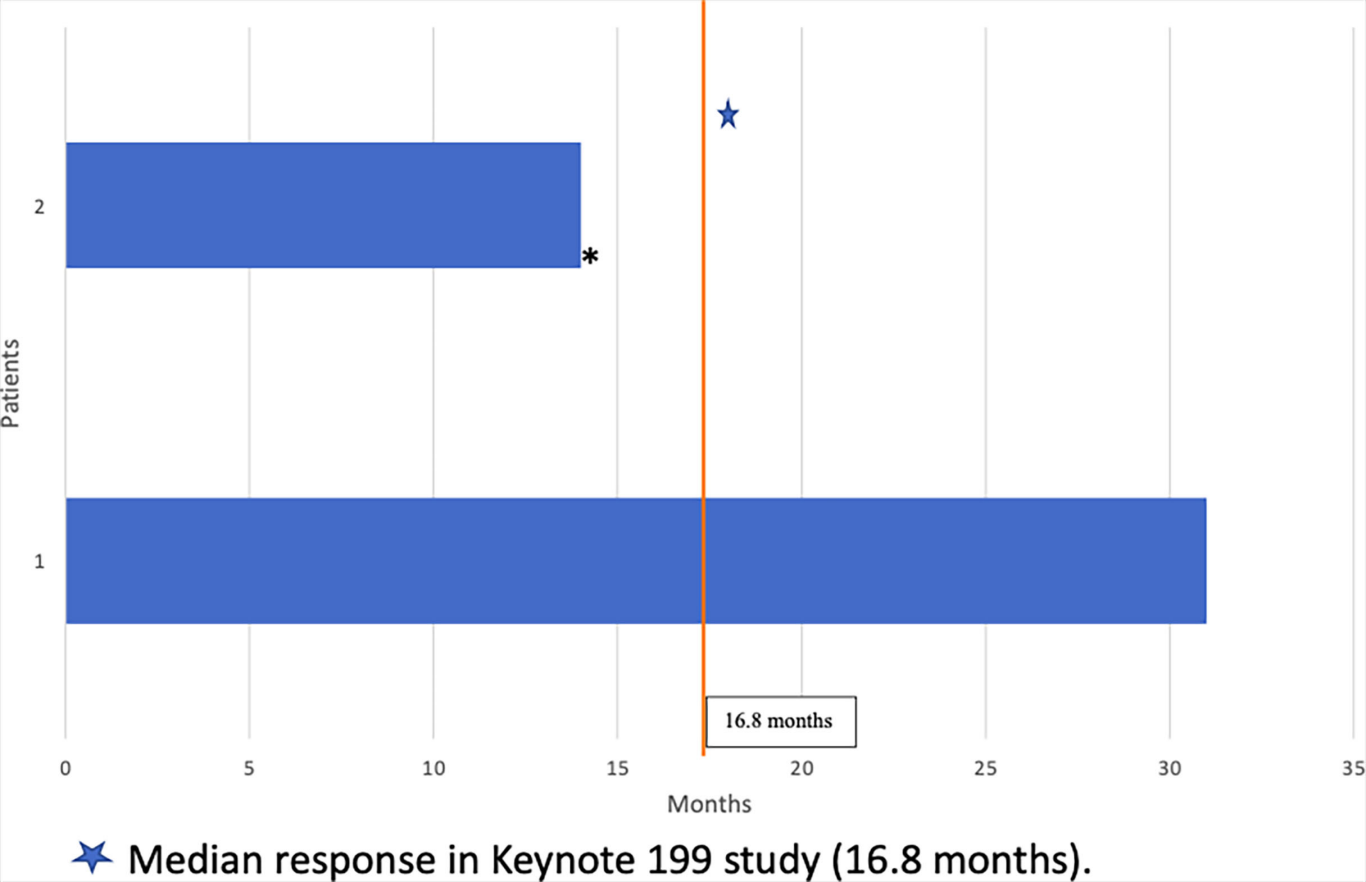
Duration of Response to Pembrolizumab (months). Median response in Keynote 199 study (16.8 months).

At the time of this analysis, one of the two patients identified was alive. While on treatment with pembrolizumab, both patients responded to therapy with biochemical and/or radiographic response. Duration of response is summarized in **Figure 2**. Individual 1 had partial response for 31 months on treatment and remains on treatment to date. Individual 2 had stable radiographic disease for 14 months prior to disease progression with new brain metastasis, clinical decline, and ultimate death a month after his last dose of pembrolizumab. Neither patient had immune-related adverse events.

## Discussion

Highly effective treatments for HIV have transformed what was once an incurable and frequently lethal condition. As the life expectancy of individuals with HIV now matches that of the general population, the cancer burden in this population will likely shift. By 2030, prostate and lung cancer are expected to emerge as the most common cancers in individuals with HIV (13). Therefore, it will be important to understand the effects of all available cancer therapies in this population.

Checkpoint inhibitors have revolutionized the treatment of many different cancers. For prostate cancer, the Keynote 199 study demonstrated the efficacy of pembrolizumab in select patients with docetaxel-refractory metastatic mCRPC (7). In that trial, the observed overall response rate was a modest 5%. However, the median duration of response among participants who achieved complete or partial response was durable at 16.8 months. Most of the participants on this trial (60%) had one or more treatment related adverse effects (TRAE), but only 15% had grade 3-5 TRAE and 5% discontinued treatment due to toxicity. Immune related adverse events (IRAE) were found in 17% of participants, with the most common iRAE being colitis, thyroid dysfunction, pneumonitis, and severe skin reactions. Notably, 2 patients died due to treatment related pneumonitis and sepsis (n=1 each) (7).

The limited case series presented here has demonstrated that some HIV positive patients can have prolonged response to pembrolizumab without increased toxicity. A prospective trial among HIV positive patients with different types of cancers has also demonstrated the safety of using pembrolizumab for this patient population (10). Because of the small sample size in this series it remains unclear whether patients with HIV and mCRPC are more likely to respond to checkpoint inhibition, and what underlying immunologic mechanisms may be operating to generate an anti-cancer immune response in these patients. Thus, it is important that future immunotherapy trials include HIV positive individuals in therapeutic clinical trials to better understand immunotherapy activity in patients who might have a lower CD4 count or higher HIV viral loads.

## Conclusions

The small case series presented in this review adds to the accumulating evidence regarding the safety and efficacy of checkpoint inhibitors in patients with HIV. In individuals with mCRPC and HIV, Pembrolizumab result in a durable response, even among those who have been heavily pretreated.

## Data Availability Statement

The original contributions presented in the study are included in the article/supplementary material, further inquiries can be directed to the corresponding author/s.

## Ethics Statement

Written informed consent was obtained from the individual(s) for the publication of any potentially identifiable images or data included in this article.

## Author Contributions

DI and HB designed the study and wrote the manuscript. TF, CP, and CR contributed to the design of the study and provided many edits to the manuscript. All authors approved the submitted manuscript

## Conflict of Interest

The authors declare that the research was conducted in the absence of any commercial or financial relationships that could be construed as a potential conflict of interest.

## Publisher’s Note

All claims expressed in this article are solely those of the authors and do not necessarily represent those of their affiliated organizations, or those of the publisher, the editors and the reviewers. Any product that may be evaluated in this article, or claim that may be made by its manufacturer, is not guaranteed or endorsed by the publisher.
